# Increasing STEM undergraduate participation in innovative activities: Field experimental evidence

**DOI:** 10.1371/journal.pone.0214155

**Published:** 2019-04-05

**Authors:** Joshua Graff Zivin, Elizabeth Lyons

**Affiliations:** 1 School of Global Policy & Strategy, University of California, San Diego, La Jolla, California, United States of America; 2 NBER, Cambridge, MA, United States of America; 3 School of Global Policy & Strategy, University of California, San Diego, La Jolla, California, United States of America; TED University, TURKEY

## Abstract

Workers trained in STEM are generally viewed as essential for innovation-led economic growth. Yet, recent statistics suggest that a majority of STEM undergraduates do not go on to pursue innovation-focused careers in their fields of study. We investigate whether STEM students who do not self-select into innovative tasks are doing so because they are less capable than their peers who do. We find that monetary inducement among STEM students increases aggregate innovative output, but that low-GPA students who were induced significantly underperform relative to low-GPA students who self-selected; however, induced and self-selected high-GPA students perform statistically the same. In contrast, words of encouragement appears to benefit those students with the lowest GPAs. Our results highlight the value of efforts to increase the pool of STEM students who pursue innovative careers and underscore the importance of interventions targeted at specific student subgroups to maximize the returns on those efforts.

## Introduction

Innovation has long been viewed as important for productivity and income growth [1, 2]. Organizational and government policy generally aims to foster innovation by targeting the quantity and quality of individuals employed in innovative careers [3]. While innovative output is not concentrated within any single industry or field [4], one potentially important target for these policies is undergraduate science, technology, engineering, and math (STEM) students who acquire a disproportionate share of the hard skills required to introduce high impact innovations into the economy [5].

Yet, despite the skills they accumulate during their Bachelor’s degrees, many STEM students do not end up in careers that require them to apply these skills to innovation. For instance, in 2014 the US Census Bureau reported that 74% of people with STEM undergraduate degrees were not employed in STEM jobs [6] despite a robust labor market in this area—nearly 2.4 million STEM job postings in the US are projected to go unfilled in 2018 alone [7]. Part of this under-supply of labor for STEM jobs is likely because STEM degrees allow graduates to pursue a range of lucrative careers that do not require them to innovate, thus making the link between STEM education and innovative output weaker than may be expected [8].

Whether efforts to encourage more of these graduates to enter innovative careers will increase the innovativeness of the economy depends fundamentally on why they are not entering such careers in the first place. Are they self-selecting out because they know they are not well-suited to the task at hand, or are other factors shaping their career choices?

In this paper, we explore whether computer science and engineering (CSE) undergraduate students can be induced to participate in an innovative task they were otherwise unwilling to participate in, and we investigate the degree to which their initial unwillingness is due to their abilities relative to their peers who did not need additional financial inducement to participate. More specifically, we explore three inter-related questions in the context of CSE students: (1) Can reluctant individuals be induced to innovate? (2) Are induced innovators less able to innovate than those who self-select into innovative activities? (3) What is the impact of encouragement on the performance of innovators, and does this differ between self-selected and induced innovators?

The interventions we implemented to investigate these research questions were undertaken within an innovation contest for undergraduate CSE students at the University of California, San Diego (UCSD). We compare self-selected innovators to a sample of induced innovators with similar technical training to test if they differ in their contest-relevant skills, and we examine their relative quantity and quality of output on an innovative task. We also assess the differential effects of confidence boosting messages across these populations to determine whether this type of encouragement intervention impacts innovative outcomes, and whether the induced innovators’ performance benefits more from these efforts.

As expected, based on observable characteristics, the induced participants were different than those who volunteered; they were less likely to be drawn from majors that provide the most relevant skills for the competition and they had lower average cumulative GPAs (CGPAs). Despite this, aggregate innovative output appears similar between the induced and self-selected participants, and we find that inducement can increase the rate of innovation. Encouragement appears to have no effect on average participant performance. Moreover, it does not appear to have a differential effect for those induced, suggesting that confidence boosting is not essential for the reluctant participants once they are induced to participate.

However, as predicted by the framework that motivates our experimental design, these average effects mask important heterogeneity in the impacts of each treatment arm. In particular, among low CGPA participants, the quality of induced participants’ innovations was significantly lower than that of self-selected participants and the submission rate among these induced participants was about half that of their self-selected peers. At the same time, students with lower classroom performance are helped by encouragement. In contrast, not only do high CGPA students not benefit from this encouragement, but they appear to be harmed by it.

Ultimately, our results suggest that it is possible to increase the proportion of STEM undergraduate students who are willing to engage in applied innovation and that this increases total innovative output. Inducements are likely to bear the most fruit when targeted toward students with high innovative potential, as proxied by information that is readily available to policy makers. The message for encouragement is less intuitive, as it appears helpful to students with lower CGPAs but harmful to those at the higher end of the academic achievement distribution.

## Materials and methods

Our experimental design and analysis are motivated by a simple and stylized framework that allows for different preferences and abilities for innovative activities. The utility function used in the framework is consistent with our setting, but intended to be general enough to be useful in other settings as well.

### Motivating framework

There are *n* students who each have an innovative type *α*. This *α* may affect a student’s cost of innovative activities, their expected likelihood of a successful innovative activity outcome, or both. For simplicity, assume there are two possible *α*’s. In reality, there is a continuum of *α* types but to be consistent with our empirical setting, this framework focuses on those at the margin of selecting into innovation. Students are presented with an opportunity to spend time on an innovative activity, referred to as an “innovation contest” that will pay them *R* if they succeed. Each student *i* succeeds with some probability *p*(*success*|*α*_*i*_) and has a cost *c*(*α*_*i*_) of spending time on the contest. Therefore, student *i*’s expected utility from participating in the innovation contest is *E*(*U*_*i*_|*α*_*i*_) = *p*(*success*|*α*_*i*_)*R* − *c*(*α*_*i*_).

Suppose that *E*(*U*_*i*_|*α*_1_) ≥ *E*(*U*_*i*_|*α*_2_) either because *p*(*success*|*α*_1_) ≥ *p*(*success*|*α*_2_), or *c*(*α*_2_) > *c*(*α*_1_), or both. The latter inequality is plausible even among very similar populations as in our empirical setting because preferences for innovating vary across individuals. In addition, suppose that *E*(*U*_*i*_|*α*_1_) ≥ *O* ≥ *E*(*U*_*i*_|*α*_2_) so that only students with *α*_1_ participate in the innovation contest. Going forward, we refer to these students as “self-selected innovators” because they are the ones that choose to voluntarily enter the contest based on their skills and costs of participation.

#### Subsidy introduction

Suppose now that those who have not selected into the innovation contest are offered some subsidy *S* that is large enough to induce participation. As a result, their expected utility from participating is now: *E*(*U*_*i*_|*α*_2_) = *p*(*success*|*α*_2_)*R* − *c*(*α*_2_) + *S* > 0. Importantly, we assume that *S* does not affect *p*(*success*|*α*_*i*_). Due to possible income effects, the subsidy is subsequently offered to *α*_1_ type students as well, albeit after they have elected to participate in the study to avoid influencing their participation decision.

If *E*(*U*_*i*_|*α*_1_) ≥ *E*(*U*_*i*_|*α*_2_) because *p*(*success*|*α*_1_) ≥ *p*(*success*|*α*_2_), and *c*(*α*_1_) is not less than *c*(*α*_2_), then self-selected innovators are more likely to succeed in the innovation contest than those innovators who were induced innovators. Alternatively, if *E*(*U*_*i*_|*α*_1_) ≥ *E*(*U*_*i*_|*α*_2_) because *c*(*α*_2_) ≥ *c*(*α*_1_), and *p*(*success*|*α*_1_) is not greater than *p*(*success*|*α*_2_), then self-selected innovators are no more likely to succeed in the innovation contest than those who were induced innovators. Although we are only allowing for one possible low *α* type in our model, in reality, it is possible that there are low *α* types who have higher costs of innovating but similar abilities to those of self-selected innovators and low *α* types who have lower likelihoods of success than self-selected innovators. We test for this possibility empirically in the Results section of the paper.

Based on this framework, our hypotheses are summarized by the following two propositions:

**Proposition 1**: If self-selected and induced innovators differ only in their probability of successfully innovating, then induced innovators should perform worse than self-selected innovators.
**Proposition 2**: If self-selected and induced innovators differ only in their cost of innovation, then, conditional on the cost of innovating being incurred, they should perform no worse on innovative activities.

These propositions motivate the “inducement” intervention in our study, in which a random subset of eligible students who do not initially sign up to participate in an innovation contest are offered a $100 subsidy, in the form of a Visa gift card, to do so.

#### Encouragement intervention

While our subsidy intervention focused on the costs of participating in innovative activities, one could also try to improve the success rate of innovators *p*(*success*|*α*_*i*_) through a series of managerial interventions. In our case, we envision a confidence boosting intervention where confidence is defined as a student’s belief in their likelihood of success in innovation. This intervention is motivated by evidence that lack confidence in abilities [9, 10] and fear of failure [11] are important barriers to pursuing STEM careers.

Suppose this intervention increases *p*(*success*|*α*_*i*_) if a student’s *i* is below some threshold. This may be the case if, for instance, lack of confidence causes poor performance due to anxiety-induced choking [12]. Moreover, to avoid generating performance harming over-confidence [13], our intervention provides encouragement rather than suggest to workers that they have exceptional skills. Planners cannot observe ex-ante which student’s are under-confident. If what differentiates *α*_1_ and *α*_2_ types is how confident in their abilities they are, then this intervention will raise *p*(*α*_2_) by more than *p*(*α*_1_).

Thus, the role of encouragement in our innovation environment can be summarized by the following proposition:

**Proposition 3**: If high and low type innovators differ in self-confidence, then interventions aimed at increasing student confidence in their abilities will increase innovative activity performance among low type innovators (*α*_2_*s*) more than it will among high type ones (*α*_1_*s*).

This proposition motivates the “encouragement” treatment in our experiment in which a random subset of students participating in the innovation contest are sent a schedule of confidence boosting emails throughout the contest period.

### Experiment design & implementation

We studied the differences between people who self-select into innovating and those who have to be induced to do so, and the effects of our encouragement treatment within an innovation contest. This contest was open to all 3,445 undergraduate students enrolled in the CSE department at UC San Diego, a top 10 globally ranked department [14] that offers “…a broad and rigorous curriculum designed to provide students with the strong academic education and technical training necessary for placement in the competitive high-tech job market as well as for advanced studies in graduate school [15].” The contest required participants to design and/or develop an application that helps people fall asleep faster individualized based on each person’s characteristics. This problem was defined through discussions with several executives and entrepreneurs in technology industries, some of whom served as contest judges. It is both an important problem that does not yet have an ideal solution, and one that we believed the undergraduate students could make reasonable progress on within a three month window. To ensure students who signed up for the contest earlier would not have a head start on the problem, students were not told about the specific problem until after the induced sample’s sign-up deadline had passed.

The contest was promoted by the UCSD CSE department over a two-month period in early 2017. Submissions made by the contest deadline were evaluated by five technology industry participants who evaluated each submission across four categories–functionality, user-friendliness, novelty, and potential commercial value–and provided a score of 1-5 on each category for a total score maximum of 20. The developers of the top three applications were awarded prize money. First place received $5,000, second place received $2,000, and third place received $1,000.

We randomly selected 1,000 students who were enrolled in CSE undergraduate majors at UCSD but who did not sign up by the original deadline to receive our inducement intervention. We over-sampled females in who we targeted for inducement because we hypothesized that they would respond differently to inducement than males. As S3 and S4 Tables demonstrate, our hypothesis was incorrect. We also over-sampled students enrolled in electrical engineering or computer science majors to mirror the sample of participants who signed-up before the original contest deadline.

Taking up the inducement offer did not require students to do anything other than put their name on the list of contest participants and agree to receive emails about the contest. Those who received our inducement offer but did not sign up for the contest in response are not in our sample; thus, we do not analyze the differences between those who take up the inducement and those who do not. In order to provide this incentive, the initial contest sign-up deadline was extended by one week. One possible concern with the inducement intervention is that those who signed up after receiving it did so because they did not see any of the contest announcements that were sent out prior to the inducement treatment. We think this is unlikely because the CSE department and the entrepreneurship center located within the department sent out numerous emails to students about the contest over a two month period. Therefore, even if the induced population did not read the emails announcing the contest, they would have chosen not to based on the subject line that made clear there was a contest being announced.

Students who had already signed up to participate (self-selected participants) were informed about the sign-up deadline extension and about the monetary incentive being offered to some students to increase the participant pool. While this disclosure was designed to avoid contamination of our treatment through ex post discovery, it is possible that informing self-selected innovators that we would be attempting to increase the participant pool could affect their effort [16]. The ultimate magnitude and direction of this change in effort, should it exist, would depend on participant priors about the initial size of the applicant pool. However, given the short sign-up extension window and the small number of induced students who signed up to participate, we believe it would have at most a very modest impact. Due to possible income effects, self-selected participants were also told they would receive the same amount of money being offered to the students who had not yet signed up. Given that those who sign up before the initial deadline were not expecting this payment so it will not bias our measure of intent. The emails sent to both the students in our inducement sample, and to the students who had self-selected into the contest are provided in S3 Fig. The innovation contest began the day after the extended contest sign-up deadline at which point the contest problem was revealed to students.

To implement our encouragement treatment, we randomly selected induced and self-selected students to receive 4 confidence boosting emails over the course of the contest. The text included in the emails differs from one email to the next, but they are all written to provide versions of messages that have been shown to correlate with employee satisfaction and productivity in organizational behavior research [17]. The complete texts of each email are included in S4 Fig.

This study was approved by the UC San Diego Institutional Review Board, project number 161649. Students were not aware that the innovation contest was part of a research study. A waiver of consent was approved for this study because the research could not be practicably implemented without the waiver. The waiver was requested primarily to prevent contributor behavioral responses due to an awareness that they were being studied. For example, students who take up the inducement offer and know that they are being studied because of it may become aware that they are different than those who self-selected into the contest in ways that affects their performance [18]. Similarly, students may respond differently to managerial interventions if they are aware they are part of a study on responses to managerial interventions. More generally, even if the details of the research study are not given to participants, they may still perform differently than if they were not aware of being observed [19].

### Empirical estimation

Given that the goal of our study is to first compare the performance of two distinct samples of innovators and second to evaluate a randomized treatment, our analysis of average performance differences focuses on mean comparisons across the induced and self-selected and the encouraged and non-encouraged groups.

We also test whether these mean differences change when individual observable differences are accounted for using regressions that control for participant characteristics and present these findings in S1 and S2 Tables. Importantly, because we expect that the primary mechanism through which induced and self-selected innovators’ performance will differ is through different motivations for selection into participation, including controls for participant characteristics eliminates important explanatory variation for performance differences between the two participant types. The encouragement treatment was randomly assigned to participants who had already selected into the contest, so we do not expect that participant characteristics will vary by encouragement treatment (comparisons are presented in the results section). As with inducement, we nonetheless run our regression analysis of the effects of encouragement on outcomes with and without controls for these characteristics.

As our framework presented above demonstrates, we expect the difference between self-selected and induced participants and the impacts of our encouragement treatment will depend on students’ innovative abilities. Using cumulative GPA as our best proxy of this ability, we assess this directly by estimating the following equation:

$$Y_{i}=\alpha_{i}+\beta_{1}\left(Intervention_{i}\right)+\beta_{2}\left(HighCGPA_{i}\right)+\beta_{3}\left(Treatment_{i}*HighCGPA_{i}\right)+\epsilon_{i}$$ (1)

where *Y*_*i*_ is a measure of performance or participation, *Intervention*_*i*_ is equal to one if participant *i* is induced or received the encouragement intervention, and *HighCGPA*_*i*_ is equal to one if participant *i* has above the sample median cumulative GPA. We estimate these equations separately for comparing induced and self-selected participants, and for evaluating the effects of the encouragement treatment.

While CGPA is far from an ideal proxy for the likelihood of successfully innovating, it is easily observable (particularly relative to more direct measures of innovative capability among students who have not previously engaged in innovation), and existing evidence demonstrates that it is correlated with factors that are also correlated with innovative capability [20].

## Results

Our contest design yielded 103 self-selected innovators—students who signed up to participate before the innovation contest sign-up deadline without inducement; and 87 induced innovators—students who only joined the contest after the provision of our financial inducement. The encouragement intervention was randomly allocated to half of the participants in each arm. Thus, our sample sizes per treatment arm are as follows: 52 participants in the self-selected, no encouragement group; 51 in the self-selected with encouragement group; 44 in the induced, no encouragement group; and 43 in the induced with encouragement group.

Data on participant degree majors, gender, year of study, GPA, and whether they have previously participated in an innovation contest were collected through surveys administered when students signed up for the contest. Our outcome measures include a simple binary variable indicating whether a project was submitted for consideration by contest judges as well as the judges’ scoring of projects that were submitted. Each project was scored by three judges. Our preferred measure of the quality of submissions is the average ranking judges gave each project. Each judge scored 7 projects, so this measure ranges from 1-7 with 7 as the highest and 1 as the lowest. However, our findings are robust to alternative measures, including normalized average scores, as shown in S5 Table.

We also ran a survey following the conclusion of the contest to better understand students’ experience with the contest. There were four versions of the survey, one for each treatment group combination, where each shared a common group of questions as well as some that were specific to treatment status. All survey questions are reported in S5 Fig. The dataset used in our analysis is publicly available through UC San Diego’s Digital Collections data repository [21].

### Average differences between induced and self-selected innovators

#### Selection into participation

Panel A of Table 1 demonstrates important differences between the induced and self-selected participant populations. Induced participants are less likely to be drawn from majors that provide the most relevant skills for the competition, and they had lower cumulative GPAs. Consistent with the predictions of our conceptual framework, inducement appears to lead students with lower probabilities of success or higher costs of participation to participate by increasing the pay-offs to participation. For instance, students with lower GPAs who agree to participate upon receiving monetary inducement may have less financial security than those who participate without the inducement, and this financial insecurity may also affect their GPA by requiring them to work outside of school.

**Table 1:** Mean participant characteristic and performance comparisons by self-selected and induced innovators.

	Self-Selected	Induced	p-value
**Panel A: Characteristics**			
Female	0.252	0.272	0.726
	(0.043)	(0.034)	
CS/EE Major	0.786	0.487	0.000***
	0.040	0.042	
Year of Study	2.941	3.111	0.365
	(0.120)	(0.146)	
CGPA (1-6)	4.500	4.274	0.096*
	(0.091)	(0.101)	
Above Median CGPA	0.663	0.521	0.065*
	(0.049)	(0.060)	
Prior Contest Experience	0.163	0.093	0.154
	(0.036)	(0.032)	
**Panel B: Outcomes**			
Average Ranking	0.390	0.264	0.460
	(0.126)	(0.108)	
Submitted Project	0.096	0.081	0.725
	(0.029)	(0.030)	
Average Ranking	4.016	3.285	0.379
Conditional on Submitting	(0.497)	(0.653)	
N	104	86	

Notes: The table compares average characteristics and innovation contest performance of self-selected and induced students. The p-values of t-tests assessing whether the means are significantly different across the two groups of students are reported in the fourth column. Standard deviations are in parentheses. Female and CS/EE Major weighted to account for over sampling in the induced treatment. CGPA is measured on a scale from 1-6 with 1 being less than 2.0, 2 being 2.0-2.49, 3 being 2.50-2.99, 4 being 3.0-3.49, 5 being 3.50-3.99, and 6 being 4.0. Average ranking is equal to the average rank assigned to the project by the three judges assigned to the project for participants who submitted a project for judgment, and zero for those who did not. Each judge scored 7 projects, so this measure ranges from 7 as the highest rank to 1 as the lowest.

* significant at 10%;

** significant at 5%;

*** significant at 1%

#### Differences in outcomes

Despite the mean comparisons suggesting that the induced participants may be less well equipped to compete, the total output of these participants appears statistically indistinguishable from the output of those that self-selected into the competition. In particular, mean submissions across induced and self-selected innovators presented in Panel B of Table 1 demonstrate that induced participants have slightly lower mean submission rates than self-selected ones but that this difference is far from statistically significant. With our sample size, we would have needed a difference in submission rate of 6 percentage points to detect a significant difference between the induced and self-selected sample which is several times larger than the 1.5 percentage points we observe. Importantly, while we cannot conclude that induced and self-selected participants have different submission rates, we can conclude that inducement increases total innovative output. Induced innovators’ likelihood of submitting a project to the contest is significantly larger than zero (p-value<0.01).

The difference in average innovative output quality conditional on submitting between induced and self-selected participants is reasonably large, with self-selected participant output scoring more than 22% higher on average than induced participant output. Although this difference is not statistically significant (with our sample size, a difference in scores of 1.172 would be statistically significant), it provides suggestive evidence that induced innovators may have lower average quality of innovative output relative to self-selected innovators.

While average outcomes are important for understanding aggregate changes in innovative output caused by increasing the pool of innovators through inducement, whether the distribution of submission quality differs for the self-selected and induced participants is also important for understanding whether inducement can lead to increases in very high impact innovation. This is particularly important for innovation management and policy because the majority of returns from innovation are generated by a small minority of innovations [22]. To investigate distributional differences in the quality of submissions, we plot the distribution of average rankings conditional on submissions for the self-selected and inducement samples (see S1 Fig). Consistent with the suggestive evidence that the quality of self-selected output is higher than that of the induced sample, it does appear that the induced sample has a higher frequency of low performance relative to the self-selected sample. At the same time, we do not find evidence that the likelihood of very high performance differs meaningfully for the two samples.

### Average effects of receiving an encouragement during the contest

Table 2 presents mean comparisons between participants who did and did not receive the encouragement treatment. As described earlier, the encouragement treatment was randomly assigned so participants who received the encouragement treatment should look similar to those who did not. Panel A on Table 2 largely confirms this with one minor exception. There is a small statistically significant difference at the 10% level in whether or not a participant is in a computer science or electrical engineering major. To ensure that this difference is not driving our findings, we confirm that all our results are robust to controlling for it in regression analyses (see S2 Table).

**Table 2:** Mean participant characteristic and performance comparisons by encouragement treatment.

	Not Encouraged	Encouraged	p-value
**Panel A: Characteristics**			
Female	0.375	0.287	0.201
	(0.050)	(0.047)	
CS/EE Major	0.760	0.648	0.093*
	(0.044)	(0.049)	
Year of Study	2.901	3.130	0.218
	(0.137)	(0.125)	
CGPA (1-6)	4.427	4.356	0.604
	(0.100)	(0.092)	
Above Median GPA	0.577	0.570	0.918
	(0.050)	(0.052)	
Prior Contest Experience	0.135	0.128	0.875
	(0.035)	(0.035)	
**Panel B: Outcomes**			
Average Ranking	0.369	0.294	0.656
	(0.118)	(0.121)	
Submitted Project	0.104	0.074	0.476
	(0.031)	(0.027)	
Average Ranking	3.583	3.904	0.702
Conditional on Submitting	(0.415)	(0.792)	
N	96	94	

Notes: The table compares average characteristics and innovation contest performance of non-encouraged and encouraged students. The p-values of t-tests assessing whether the means are significantly different across the two groups of students are reported in the fourth column. Standard deviations are in parentheses. Female and CS/EE Major weighted to account for over sampling in the induced treatment. CGPA is measured on a scale from 1-6 with 1 being less than 2.0, 2 being 2.0-2.49, 3 being 2.50-2.99, 4 being 3.0-3.49, 5 being 3.50-3.99, and 6 being 4.0. Average ranking is equal to the average rank assigned to the project by the three judges assigned to the project for participants who submitted a project for judgment, and zero for those who did not. Each judge scored 7 projects, so this measure ranges from 7 as the highest rank to 1 as the lowest.

* significant at 10%;

** significant at 5%;

*** significant at 1%

Panel B of Table 2 presents mean outcomes by encouragement treatment status. Interestingly, encouragement does not appear to have a consistent or significant impact on participant outcomes in the contest. For submission rates to have been significantly different between participants who received the encouragement treatment and those who did not with our sample size, we would have needed to observe a difference of about 5.5 percentage points (or almost twice the size of what we find). Similarly, we would have needed to observe a mean average ranking difference of about 0.95, more than twice as big as the difference we observe, for submission quality to have been significantly different with our sample size.

Moreover, as reported in S6 Table, we do not find any differential impacts of encouragement across the induced and self-selected populations as described in Proposition 3, suggesting that lack of confidence may not be the primary explanation for why some students select out of innovating. Consistent with no significant average effects of encouragement or inducement on performance, we do not find a relationship between self-reported effort on the contest and whether a participant is induced or encouraged (see S12 Table).

We also investigate whether encouragement led to a change in the quality distribution of submissions by plotting the distribution of average rankings conditional on submissions for the encouraged and non-encouraged samples (S2 Fig). The evidence from this graph demonstrates that submissions made by those in the encouraged sample have a higher frequency of very low or very high performance, and that the encouraged sample is about twice as likely to have an average rank of at least 4 (the sample median score) than the non-encouraged sample. While these differences are not statistically significant, to the extent that firms mostly value output in the upper tail of the quality distribution, they provide some suggestive evidence that encouragement may be beneficial.

### Heterogeneity in average outcomes by GPA

We theorized in our motivating framework that the presence of performance differences between induced and self-selected participants would depend on the characteristics of the individuals drawn into the competition. In particular, induced participants should do worse than their self-selected counterparts if the initially reluctant students are drawn from the lower end of the probability of innovative success distribution. If, on the other hand, the induced participants were initially on the sidelines simply because their costs of participating were higher than those of their self-selected counterparts then they should not perform worse in the contest. In reality, our inducement intervention could draw in both types of individuals.

**Fig 1:**
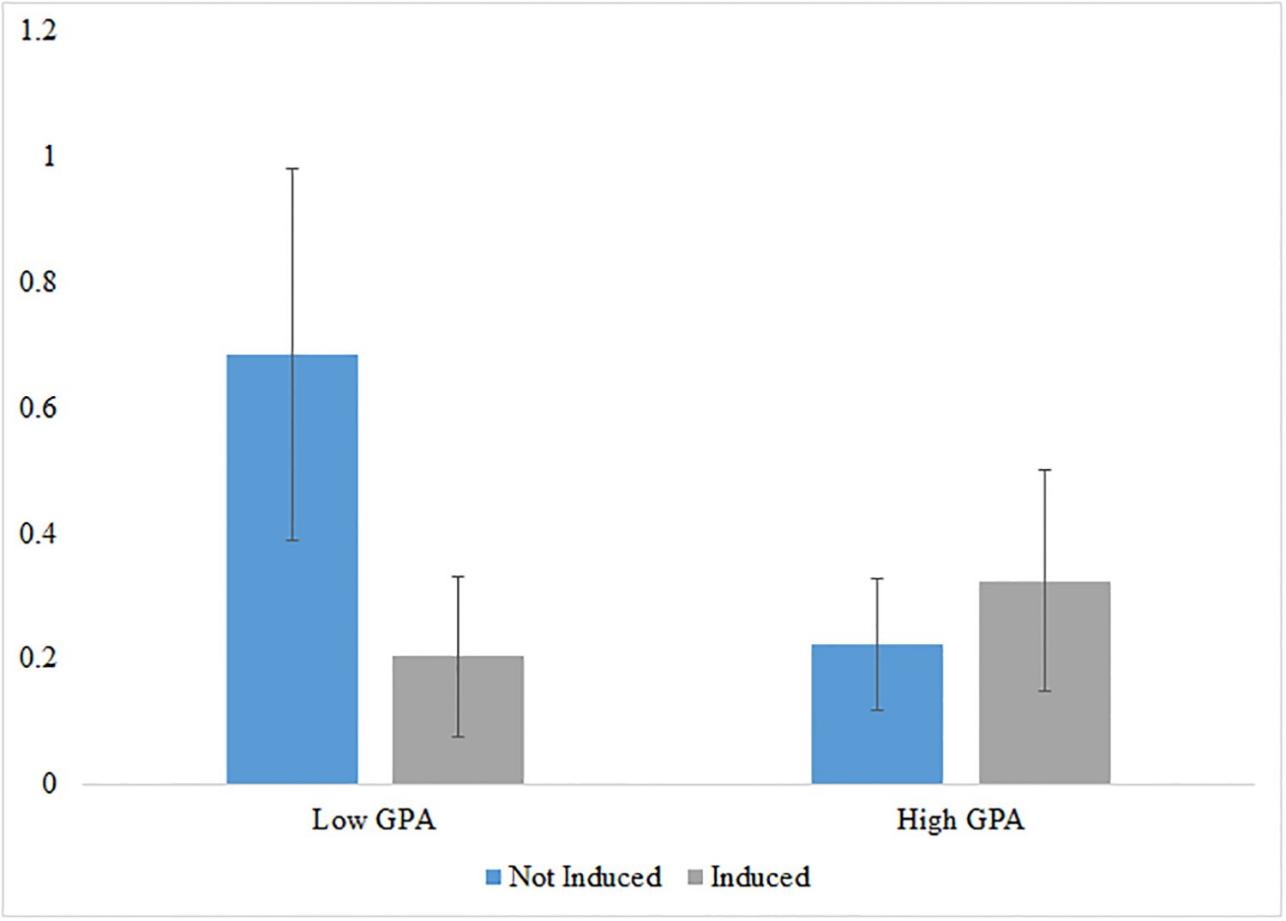
Difference in average ranking by self-selected and induced participants and GPA. The figure compares average contest performance across below and above median CGPA students, and self-selected and induced students. Standard errors are displayed as black bars. Average ranking is equal to the average rank assigned to the project by the three judges assigned to the project for participants who submitted a project for judgment, and zero for those who did not. Each judge scored 7 projects, so this measure ranges from 7 as the highest rank to 1 as the lowest.

Fig 1 examines these differential effects in greater detail. It presents the difference in mean contest performance between induced and self-selected contest participants for below- and above-median CGPA participants respectively. These means support our predictions that when inducement leads individuals who are less capable of innovating to participate in innovation, it leads to lower innovative performance. In particular, induced students with low GPAs perform significantly worse, about 250 percent worse, in the contest than their self-selected peers, whereas high-GPA participants who were induced perform statistically the same as those who self-selected into the contest. These findings are robust to regression analysis with interactions between GPA and inducement and controls for participant characteristics as demonstrated in S7 Table.

Interestingly, the highest performers are self-selected low-GPA students suggesting that they may have private information about their innovative capabilities that are not reflected in their academic performance.

**Fig 2:**
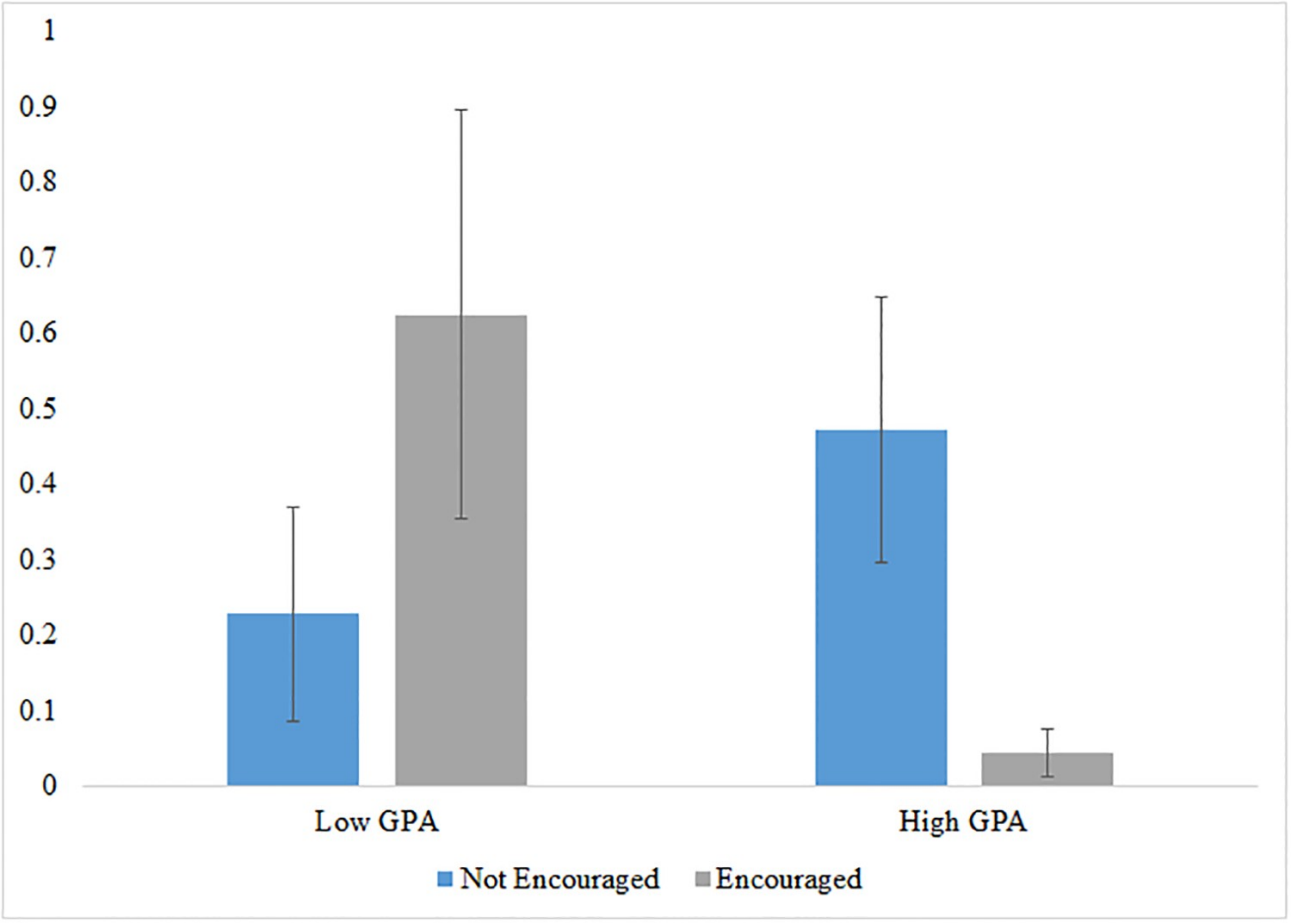
Change in average ranking due to encouragement by GPA. The figure compares average contest performance across below and above median CGPA students, and those who received the encouragement treatment and those who did not. Standard errors are displayed as black bars. Average ranking is equal to the average rank assigned to the project by the three judges assigned to the project for participants who submitted a project for judgment, and zero for those who did not. Each judge scored 7 projects, so this measure ranges from 7 as the highest rank to 1 as the lowest.

Recall from Proposition 3, that encouragement may have differential effects based on students’ innovative abilities. For example, lower-ability participants may be less confident than high-ability ones, with encouragement disproportionately helping the former. Fig 2 examines this directly. It presents the difference in mean contest performance between encouraged and not encouraged for participants below and above the median CGPA for all participants. Low-GPA students benefit from the additional support provided by encouragement—performing about three times better than their non-encouraged peers. The impacts on high GPA students are quite surprising. Not only do they not benefit from this encouragement, but they appear to be harmed by it. Non-encouraged high GPA participants perform five times better than those who were encouraged. These findings are robust to regression analysis with interactions between GPA and encouragement and controls for participant characteristics as demonstrated in S10 Table.

Given our initial hypothesis that our encouragement intervention would impact the self-selected and induced populations differently, we also examine whether encouragement affects contest performance among high and low GPA students differently depending on whether they are induced or self-selected. We do not find any evidence of differential impacts of encouragement across these sub-samples (see S11 Table).

Evidence from our post-contest survey responses suggests one reason our encouragement intervention harms the performance of high GPA students—it appears to increase the salience of the time commitment required for the contest. In particular, we find that the encouragement treatment is associated with an approximately thirty percentage point increase in the likelihood that participants report not submitting a project for consideration by the judges due to time constraints. By contrast we find no relationship between being induced or encouraged and participants reporting that they did not submit due to the difficulty of the contest problem. S12 Table reports these estimates. Whether the negative effect of encouragement on high GPA students also relates to the crowding out of intrinsic motivation remains an open question [23].

## Conclusion

Our study provides novel evidence that some STEM students may be selecting out of innovative activities based on their expected performance, while others select out based on the costs of participating. The results demonstrate that innovators can be created by subsidizing their initial entry into innovative tasks, but that targeting inducement towards those who select out due to their expected cost of participation rather than their expected performance is a more effective strategy to promote innovation. That such targeting can be based on relatively easy information to obtain, like training and GPA, suggests that such a strategy may be both practical and cost effective. In addition, we demonstrate that encouragement may also need to be targeted to improve the performance of those students who stand to benefit the most from it, and importantly, to avoid harming those who may respond negatively.

Several important caveats of our study are worth highlighting. First, the sample of students who submitted a project for consideration is quite low. Although we have anecdotal evidence that a 10% submission rate is standard for this type of competition, thus bolstering the external validity of our findings, some of our insignificant results may be in part due to lack of statistical power. Second, while our encouragement emails were motivated by evidence on motivating employees [17], there are many ways to provide students with encouragement aimed at enhancing performance. Accordingly, it remains possible that alternative encouragement interventions could avoid the detrimental effects on the performance of high GPA students while still improving outcomes for those with lower GPAs. Third, our measures of contest success are based on quantifiable performance metrics provided by expert evaluators rather than market-based measures of success.

Lastly, how well our findings generalize to entry into and performance for those in innovative careers likely depends on the specific characteristics of those careers. Innovation is clearly not limited to STEM fields. In our study, we have have attempted to make our contest as similar as possible to the types of innovation tasks a STEM graduate might face in an innovative job by setting the problem while providing limited guidance on requirements for the solution [24]. Moreover, many technology companies run innovation contests for their employees to encourage innovation (see https://devpost.com/ for examples). At the same time, employees in a company typically have managers they can turn to with technical or design questions, and they likely have other social and technical resources that participants in our contest did not have access to. Investigating whether these resources change the performance of some or all innovator types, particularly those who need an additional nudge to enter these careers, is an important area for future research.

## Supporting information

S1 Fig Average judge ranking by induced and self-selected samples. The figure compares the distribution of contest performance across the induced and self-selected sample. Average ranking is equal to the average rank assigned to the project by the three judges assigned to the project for participants who submitted a project for judgment, and zero for those who did not. Each judge scored 7 projects, so this measure ranges from 7 as the highest rank to 1 as the lowest.
(PDF)

S2 Fig Average judge ranking by encouraged and non-encouraged. The figure compares the distribution of contest performance across the induced and self-selected sample. Average ranking is equal to the average rank assigned to the project by the three judges assigned to the project for participants who submitted a project for judgment, and zero for those who did not. Each judge scored 7 projects, so this measure ranges from 7 as the highest rank to 1 as the lowest.
(PDF)

S3 Fig Inducement emails. (PDF)

S4 Fig Encouragement emails. (PDF)

S5 Fig Post-contest survey. (PDF)

S1 Table Difference in outcomes for induced and self-selected innovators. Standard errors are in parentheses. Columns 2, 4, and 6 include controls for participant gender, CGPA, year of study, whether or not they major in computer science or electrical engineering, and whether or not they have prior innovation contest experience. * significant at 10%; ** significant at 5%; *** significant at 1%.
(PDF)

S2 Table Effect of encouragement treatment on contest outcomes. Standard errors are in parentheses. Columns 2, 4, and 6 include controls for participant gender, cgpa, year of study, whether or not they major in computer science or electrical engineering, and whether or not they have prior innovation contest experience. * significant at 10%; ** significant at 5%; *** significant at 1%.
(PDF)

S3 Table Difference in outcomes for induced and self-selected innovators by gender. Standard errors are in parentheses. * significant at 10%; ** significant at 5%; *** significant at 1%.
(PDF)

S4 Table Effects of encouragement on contest outcomes by gender. Standard errors are in parentheses. * significant at 10%; ** significant at 5%; *** significant at 1%.
(PDF)

S5 Table Alternate measures of performance. Standard errors are in parentheses. * significant at 10%; ** significant at 5%; *** significant at 1%.
(PDF)

S6 Table Interaction between induced innovators & encouragement treatment. Tandard errors are in parentheses. Columns 2, 4, and 6 include controls for participant gender, cgpa, year of study, whether or not they major in computer science or electrical engineering, and whether or not they have prior innovation contest experience. * significant at 10%; ** significant at 5%; *** significant at 1%.
(PDF)

S7 Table Difference in outcomes for induced and self-selected innovators by GPA. Standard errors are in parentheses. * significant at 10%; ** significant at 5%; *** significant at 1%.
(PDF)

S8 Table Difference in outcomes for induced and self-selected innovators by GPA controls. Standard errors are in parentheses. All columns include controls for participant gender, year of study, whether or not they major in computer science or electrical engineering, and whether or not they have prior innovation contest experience.
(PDF)

S9 Table Effect of encouragement treatment by GPA. Standard errors are in parentheses. * significant at 10%; ** significant at 5%; *** significant at 1%.
(PDF)

S10 Table Effect of encouragement treatment by GPA, controls. Tandard errors are in parentheses. All columns include controls for participant gender, year of study, whether or not they major in computer science or electrical engineering, and whether or not they have prior innovation contest experience.
(PDF)

S11 Table Difference in outcomes for induced and self-selected participants by encouragement and GPA. Standard errors are in parentheses. * significant at 10%; ** significant at 5%; *** significant at 1%.
(PDF)

S12 Table Survey outcomes by induced participants and encouragement treatment. Standard errors are in parentheses. All columns include controls for participant gender, cgpa, year of study, whether or not they major in computer science or electrical engineering, and whether or not they have prior innovation contest experience.
(PDF)
