# Supplementary material for: Increasing STEM undergraduate participation in innovative activities: Field experimental evidence
Source: PLoS One. 2019 Apr 5;14(4):e0214155. doi: 10.1371/journal.pone.0214155 (PMC6450611; doi:10.1371/journal.pone.0214155)
Supplement: S1 Fig — The figure compares the distribution of contest performance across the induced and self-selected sample. Average ranking is equal to the average rank assigned to the project by the three judges assigned to the project for participants who submitted a project for judgment, and zero for those who did not. Each judge scored 7 projects, so this measure ranges from 7 as the highest rank to 1 as the lowest. (PDF) [file pone.0214155.s001.pdf]

Figure S1: Average Judge Ranking by Induced and Self-Selected Samples

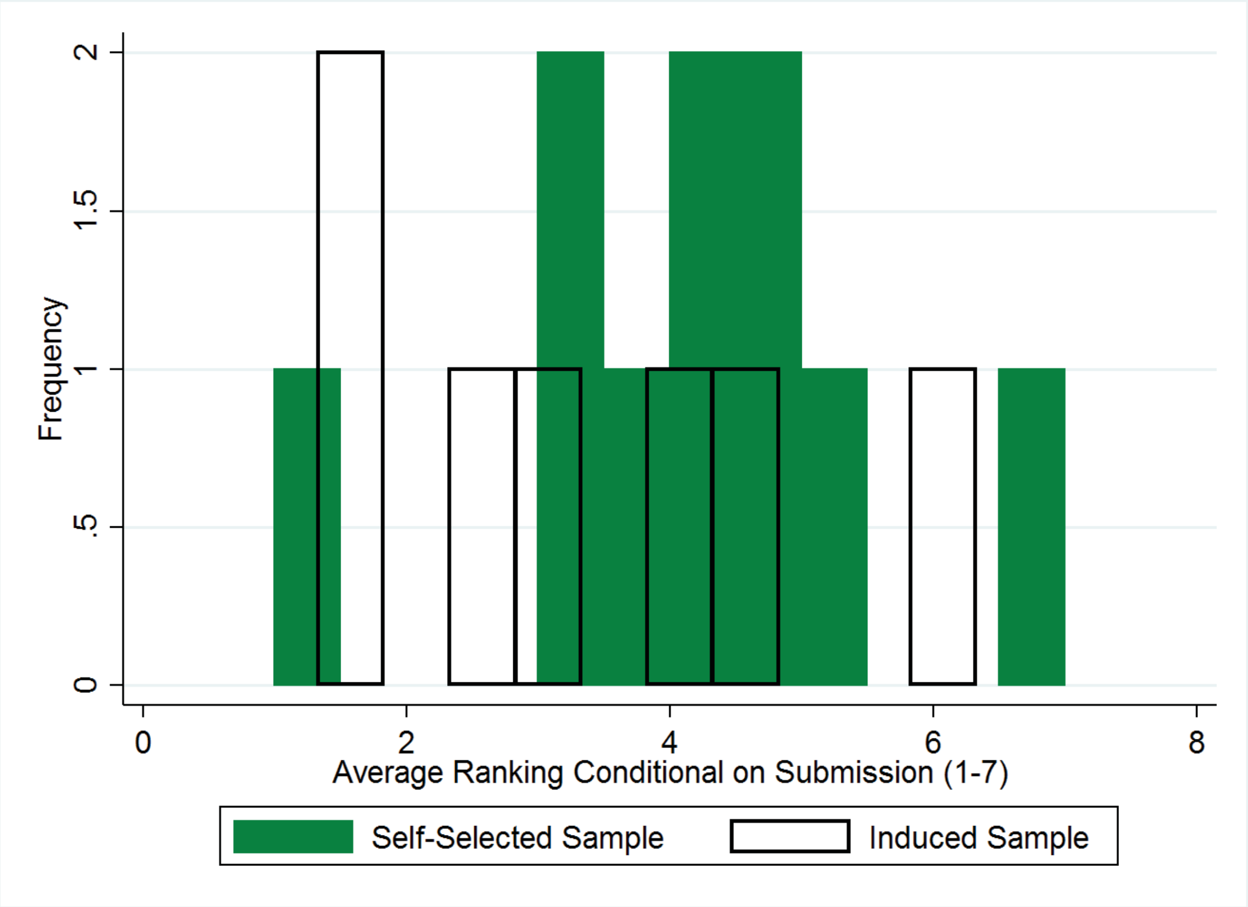

Notes: The figure compares the distribution of contest performance across the induced and self-selected sample. Average ranking is equal to the average rank assigned to the project by the three judges assigned to the project for participants who submitted a project for judgment, and zero for those who did not. Each judge scored 7 projects, so this measure ranges from 7 as the highest rank to 1 as the lowest.
