# Supplementary material for: Increasing STEM undergraduate participation in innovative activities: Field experimental evidence
Source: PLoS One. 2019 Apr 5;14(4):e0214155. doi: 10.1371/journal.pone.0214155 (PMC6450611; doi:10.1371/journal.pone.0214155)
Supplement: S3 Fig — (PDF) [file pone.0214155.s003.pdf]

Inducement Emails

The email offering a monetary incentive for contest participation sent to students offered the inducement is copied below.

Hello,
The 2017 UC San Diego Student Innovation Contest is offering students the opportunity to solve a real world problem and win up to $5,000! Thanks to an award from the Kauffman Foundation, the contest organizers are offering a $100 pre-paid visa card for participating in the contest. A random sample of students have been selected for this invitation-only opportunity and you are one of them.

The organizers have postponed the sign up deadline from February 1 until February 7 to allow students who receive this invitation to take advantage of this opportunity. If you would like to participate in the contest, please sign up by 6 pm on February 7 and you will automatically be awarded a $100 visa card. For more information and to sign up for the contest, please visit: *contest website*.

This contest is open to all Jacobs School of Engineering undergraduate students.

Sincerely,
Contest organizer

The email explaining the monetary incentive and sign-up deadline extension to students in the self-selected group is copied below.

Hello,

Thank you for signing up to participate in the 2017 UC San Diego Student Innovation Contest. Thanks to a generous award from the Kauffman Foundation, the contest organizers are offering students a $100 pre-paid visa card just for participating in the contest! In addition, they have invited a randomly selected set of students to sign up and also take advantage of this opportunity. To allow these students time to sign up, the organizers have postponed the start of the contest until February 8.

As a result of these changes, you will be receiving a $100 visa card, and a description of the problem you are to solve for the contest will be announced on February 8. The organizers apologize for any inconveniences this delay may cause you. Details on how to collect your visa card will be sent to you within the next three weeks.

Sincerely,
Contest organizer
