# Supplementary material for: Increasing STEM undergraduate participation in innovative activities: Field experimental evidence
Source: PLoS One. 2019 Apr 5;14(4):e0214155. doi: 10.1371/journal.pone.0214155 (PMC6450611; doi:10.1371/journal.pone.0214155)
Supplement: S4 Fig — (PDF) [file pone.0214155.s004.pdf]

Encouragement Emails

Encouragement Email #1

Dear UCSD Student Innovation Contest Participant,

We hope that youre enjoying the Quarter! We wanted to reach out to tell you how happy we are that you are participating in the first ever UCSD Student Application Innovation Contest. We are confident that you will enjoy the time you spend developing your submission, and that you will gain valuable experience and knowledge through the process. By developing and refining your creativity, technical abilities, and project management skills we strongly believe that this contest will prepare you for a rewarding and innovative career. In addition, the solution you are developing for how an app can be used to help people fall asleep has the potential to have meaningful social and commercial value.

We also want to remind you that UCSD has a number of resources available for students interested in furthering their innovation capabilities, including the Institute for the Global Entrepreneur at the Jacobs School of Engineering (http://jacobsschool.ucsd.edu/globalentrepreneur/).

We look forward to seeing your innovation!

Sincerely,
Contest Organizers

Encouragement Email #2

Dear UCSD Student Innovation Contest Participant,

You are now almost one third of the way through the first UCSD Student Innovation Contest! This also means that you still have over ten weeks to work on your submission. We hope that you have begun to make progress on your solution for helping people fall asleep faster, but if you havent had the chance to work on it yet, there is plenty of time remaining to develop a solution in time for the deadline.

We look forward to seeing your innovation!

Sincerely,
Contest Organizers

Encouragement Email #3

Dear UCSD Student Innovation Contest Participant,

We hope youre enjoying Spring Break and getting some time to do things you enjoy! The contest judges and organizers are very excited about the amount of creativity, effort, and knowledge being put into finding a solution to help people fall asleep faster and are looking forward to seeing your proposals and applications. Just as a reminder, UCSD has some excellent resources for students considering careers in entrepreneurship and innovation, including the Institute for the Global Entrepreneur at the Jacobs School of Engineering (http://jacobsschool.ucsd.edu/globalentrepreneur/) and the Office of Research Affairs (http://innovation.ucsd.edu/entrepreneur/).

We look forward to seeing your innovation!

Sincerely,
Contest Organizers

Encouragement Email #4

Dear UCSD Student Innovation Contest Participant,

We hope your Spring Quarter has gotten off to a good start! We have just over 5 weeks left until the contest deadline plenty of time for you to come up with and improve your solution to helping people fall asleep faster with an application. As a student in one of the 20 most innovative Computer Science and Engineering departments in the US, we are thrilled to have you working on an application that has the potential to have an impact on peoples well-being, and generate commercial success as well. In addition to the sources available to students interested in careers in innovation and entrepreneurship (http://jacobsschool.ucsd.edu/globalentrepreneur/, (http://innovation.ucsd.edu/entrepreneur/), the Kauffman Foundation, one of the contest sponsors, also has some great resources you can take advantage of (http://www.kauffman.org).

We look forward to seeing your innovation!

Sincerely,
Contest Organizers
