# Supplementary material for: Increasing STEM undergraduate participation in innovative activities: Field experimental evidence
Source: PLoS One. 2019 Apr 5;14(4):e0214155. doi: 10.1371/journal.pone.0214155 (PMC6450611; doi:10.1371/journal.pone.0214155)
Supplement: S5 Fig — (PDF) [file pone.0214155.s005.pdf]

Post-Contest Survey

UCSD Application Student Innovation Contest Post-Contest Participant Survey

* Required

1. **Why did you not sign up for the contest until you received the $100 gift card offer to sign up? ***

   Mark only one oval.

   ◯ I was not aware of the contest until I was notified that I had been randomly selected to receive $100 for participating
   ◯ I forgot to sign up before the initial contest deadline
   ◯ I did not think that I had time to participate
   ◯ I did not think that I had the abilities required to participate in the innovation contest
   ◯ I did not think the innovation contest was interesting or worth my time
   ◯ Other: ____________________

2. **Did you spend any time working on a solution for the innovation contest problem? ***

   Mark only one oval.

   ◯ Yes *Skip to question 4.*
   ◯ No

3. **Why didn't you spend any time working on a solution for the innovation contest problem? Check all options that apply.**

   Check all that apply.

   ☐ Classes took too much of my time
   ☐ Work took too much of my time
   ☐ My social life took too much of my time
   ☐ I didn't know how to begin working on the problem
   ☐ I didn't have any ideas about how to solve the problem
   ☐ I never intended to spend any time on the contest
   ☐ I didn't think the contest problem was interesting enough to work on
   ☐ Other: ____________________

Skip to question 6.

4. **Did you submit a project for consideration by the contest judges? ***

   Mark only one oval.

   ◯ Yes *Skip to question 6.*
   ◯ No

5. **Why didn't you submit a project for consideration by the judges? Check all options that apply** *

Check all that apply.

- [ ] Project proved harder than I expected and I did not have enough time to finish it
- [ ] I had more school work than expected and I did not have enough time to finish my project
- [ ] I didn't think my solution was good enough to submit
- [ ] I was worried about the judges thinking poorly of my submission
- [ ] I didn't know how to submit my project
- [ ] I forgot when the deadline for submissions was
- [ ] I lost interest in the contest
- [ ] Other

6. **Do you think you made the right decision by signing up to participate in the UCSD Student Innovation contest?** *

Mark only one oval.

- ◯ Yes
- ◯ No

7. **Did the check-in emails you received from contest organizers have any of the following impacts? Check all that apply** *

Check all that apply.

- [ ] They reminded me to work on the project
- [ ] They provided me with useful information on how to work on the project
- [ ] They made me feel more confident about my ability to do a good job
- [ ] They made me feel less confident about my ability to do a good job
- [ ] They annoyed me or wasted my time
- [ ] They made me feel less motivated to work on the project
- [ ] They made me feel more motivated to work on the project
- [ ] They made me feel supported by the contest organizers
- [ ] They had no impact on me
- [ ] Other: ____________________

8. **How would you rate your experience with the UCSD Student Innovation contest?** *

Mark only one oval.

	1	2	3	4	5	
Very negatively	◯	◯	◯	◯	◯	Very positively

9. **Will you consider participating in another innovation contest?** *
Mark only one oval.

- Yes
- No
- Maybe

10. **How could we improve the contest going forward?** *

Please provide your contact information so that you can be included in the draw to win one of 50 $100 Visa Gift Cards

11. **Last Name**

12. **First Name**

13. **UCSD Email Address**

Notes: Question 1 was only included in the survey given to the induced population. Question 7 was only included in the survey given to the encouraged population.
