# Supplementary material for: Increasing STEM undergraduate participation in innovative activities: Field experimental evidence
Source: PLoS One. 2019 Apr 5;14(4):e0214155. doi: 10.1371/journal.pone.0214155 (PMC6450611; doi:10.1371/journal.pone.0214155)
Supplement: S1 Table — Standard errors are in parentheses. Columns 2, 4, and 6 include controls for participant gender, CGPA, year of study, whether or not they major in computer science or electrical engineering, and whether or not they have prior innovation contest experience. * significant at 10%; ** significant at 5%; *** significant at 1%. (PDF) [file pone.0214155.s006.pdf]

Table S1: Difference in Outcomes for Induced and Self-Selected Innovators

	(1)	(2)	(3)	(4)	(5)	(6)
	Submission		Average Ranking		Average Ranking Conditional on Submitting	
Induced	-0.017	-0.024	-0.126	-0.190	-0.732	-0.527
	(0.0418)	(0.0475)	(0.170)	(0.191)	(0.806)	(0.846)
Controls	No	Yes	No	Yes	No	Yes
Observations	190	172	190	172	17	17
R-squared	0.001	0.025	0.003	0.046	0.052	0.404
Mean dep var	0.090	0.090	0.510	0.510	2.824	2.824

Notes: Standard errors are in parentheses. Columns 2, 4, and 6 include controls for participant gender, CGPA, year of study, whether or not they major in computer science or electrical engineering, and whether or not they have prior innovation contest experience. * significant at 10%; ** significant at 5%; *** significant at 1%
