# Supplementary material for: Increasing STEM undergraduate participation in innovative activities: Field experimental evidence
Source: PLoS One. 2019 Apr 5;14(4):e0214155. doi: 10.1371/journal.pone.0214155 (PMC6450611; doi:10.1371/journal.pone.0214155)
Supplement: S2 Table — Standard errors are in parentheses. Columns 2, 4, and 6 include controls for participant gender, cgpa, year of study, whether or not they major in computer science or electrical engineering, and whether or not they have prior innovation contest experience. * significant at 10%; ** significant at 5%; *** significant at 1%. (PDF) [file pone.0214155.s007.pdf]

Table S2: Effect of Encouragement Treatment on Contest Outcomes

	(1)	(2)	(3)	(4)	(5)	(6)
	Submission		Average Ranking		Average Ranking Conditional on Submitting	
Encouragement	-0.028	-0.020	-0.075	-0.014	0.322	0.425
	(0.042)	(0.047)	(0.169)	(0.190)	(0.824)	(0.948)
Controls	No	Yes	No	Yes	No	Yes
Observations	190	172	190	172	17	17
R-squared	0.002	0.025	0.001	0.040	0.010	0.393
Mean dep var	0.0895	0.0895	0.510	0.510	2.824	2.824

Notes: Standard errors are in parentheses. Columns 2, 4, and 6 include controls for participant gender, cgpa, year of study, whether or not they major in computer science or electrical engineering, and whether or not they have prior innovation contest experience. * significant at 10%; ** significant at 5%; *** significant at 1%
