# Supplementary material for: Increasing STEM undergraduate participation in innovative activities: Field experimental evidence
Source: PLoS One. 2019 Apr 5;14(4):e0214155. doi: 10.1371/journal.pone.0214155 (PMC6450611; doi:10.1371/journal.pone.0214155)
Supplement: S3 Table — Standard errors are in parentheses. * significant at 10%; ** significant at 5%; *** significant at 1%. (PDF) [file pone.0214155.s008.pdf]

Table S3: Difference in Outcomes for Induced and Self-Selected Innovators by Gender

	(1) Submission	(2) Average Ranking	(3) Average Ranking Conditional on Submitting
Induced	-0.011	-0.087	-0.607
	(0.052)	(0.212)	(1.002)
Female	0.024	0.276	1.643
	(0.065)	(0.265)	(1.103)
Inducemed *	-0.023	-0.203	-0.783
Female	(0.090)	(0.366)	(1.645)
Observations	190	190	17
R-squared	0.002	0.009	0.216
Mean dep var	0.0895	0.332	3.715

Notes: Standard errors are in parentheses. * significant at 10%; ** significant at 5%; *** significant at 1%
