# Supplementary material for: Increasing STEM undergraduate participation in innovative activities: Field experimental evidence
Source: PLoS One. 2019 Apr 5;14(4):e0214155. doi: 10.1371/journal.pone.0214155 (PMC6450611; doi:10.1371/journal.pone.0214155)
Supplement: S4 Table — Standard errors are in parentheses. * significant at 10%; ** significant at 5%; *** significant at 1%. (PDF) [file pone.0214155.s009.pdf]

Table S4: Effects of Encouragement on Contest Outcomes by Gender

	(1) Submission	(2) Average Ranking	(3) Average Ranking Conditional on Submitting
Encouragement	0.009	0.046	0.189
	(0.051)	(0.207)	(0.909)
Female	0.057	0.289	0.767
	(0.060)	(0.246)	(0.949)
Encouragement*	-0.111	-0.337	2.845
Female	(0.089)	(0.363)	(1.878)
Observations	190	190	17
R-squared	0.011	0.009	0.309
Mean dep var	0.0895	0.332	3.715

Notes: Standard errors are in parentheses. * significant at 10%; ** significant at 5%; *** significant at 1%
