# Supplementary material for: Increasing STEM undergraduate participation in innovative activities: Field experimental evidence
Source: PLoS One. 2019 Apr 5;14(4):e0214155. doi: 10.1371/journal.pone.0214155 (PMC6450611; doi:10.1371/journal.pone.0214155)
Supplement: S5 Table — Standard errors are in parentheses. * significant at 10%; ** significant at 5%; *** significant at 1%. (PDF) [file pone.0214155.s010.pdf]

Table S5: Alternate Measures of Performance

	(1) Average Ranking (IHS)	(2) Average Normalized Score	(3) Average Normalized Score Conditional on Submitting	(4) Average Normalized Score (IHS)
Induced	-0.098	-0.084	-0.327	-0.059
	(0.128)	(0.134)	(0.773)	(0.091)
Encouragement	-0.028	-0.031	0.473	-0.013
	(0.128)	(0.133)	(0.773)	(0.091)
Observations	190	190	17	190
R-squared	0.003	0.002	0.047	0.002
Mean dep var	0.582	0.253	2.824	0.510

Notes: Standard errors are in parentheses. * significant at 10%; ** significant at 5%; *** significant at 1%
