# Supplementary material for: Increasing STEM undergraduate participation in innovative activities: Field experimental evidence
Source: PLoS One. 2019 Apr 5;14(4):e0214155. doi: 10.1371/journal.pone.0214155 (PMC6450611; doi:10.1371/journal.pone.0214155)
Supplement: S6 Table — Tandard errors are in parentheses. Columns 2, 4, and 6 include controls for participant gender, cgpa, year of study, whether or not they major in computer science or electrical engineering, and whether or not they have prior innovation contest experience. * significant at 10%; ** significant at 5%; *** significant at 1%. (PDF) [file pone.0214155.s011.pdf]

Table S6: Interaction between Induced Innovators & Encouragement Treatment

	(1)	(2)	(3)	(4)	(5)	(6)
	Submission		Average Ranking		Average Ranking Conditional on Submitting	
Induced	0.019	0.016	0.031	-0.019	-0.367	-0.253
	(0.059)	(0.066)	(0.238)	(0.267)	(1.099)	(1.180)
Encouragement	0.006	0.018	0.071	0.151	0.501	0.683
	(0.057)	(0.064)	(0.230)	(0.259)	(1.099)	(1.308)
Encouragement*	-0.073	-0.079	-0.318	-0.344	-0.902	-0.729
Induced	(0.084)	(0.093)	(0.340)	(0.374)	(1.823)	(2.053)
Controls	No	Yes	No	Yes	No	Yes
Observations	190	172	190	172	17	17
R-squared	0.007	0.030	0.009	0.051	0.072	0.424
Mean dep var	0.0895	0.0895	0.510	0.510	2.824	2.824

Notes: Standard errors are in parentheses. Columns 2, 4, and 6 include controls for participant gender, cgpa, year of study, whether or not they major in computer science or electrical engineering, and whether or not they have prior innovation contest experience. * significant at 10%; ** significant at 5%; *** significant at 1%
