# Supplementary material for: Increasing STEM undergraduate participation in innovative activities: Field experimental evidence
Source: PLoS One. 2019 Apr 5;14(4):e0214155. doi: 10.1371/journal.pone.0214155 (PMC6450611; doi:10.1371/journal.pone.0214155)
Supplement: S7 Table — Standard errors are in parentheses. * significant at 10%; ** significant at 5%; *** significant at 1%. (PDF) [file pone.0214155.s012.pdf]

Table S7: Difference in Outcomes for Induced and Self-Selected Innovators by GPA

	(1) Submission	(2) Average Ranking	(3) Average Ranking Conditional on Submitting
Induced	-0.067	-0.480*	-2.068*
	(0.064)	(0.259)	(1.085)
Above Median CGPA	-0.059	-0.460*	-2.101**
	(0.059)	(0.238)	(0.939)
Above Median CGPA* Induced	0.084	0.581*	2.601
	(0.085)	(0.344)	(1.473)
Observations	190	190	17
R-squared	0.007	0.024	0.323
Mean dep var	0.09	0.332	3.715

Notes: Standard errors are in parentheses. * significant at 10%; ** significant at 5%; *** significant at 1%
