# Supplementary material for: Increasing STEM undergraduate participation in innovative activities: Field experimental evidence
Source: PLoS One. 2019 Apr 5;14(4):e0214155. doi: 10.1371/journal.pone.0214155 (PMC6450611; doi:10.1371/journal.pone.0214155)
Supplement: S8 Table — Standard errors are in parentheses. All columns include controls for participant gender, year of study, whether or not they major in computer science or electrical engineering, and whether or not they have prior innovation contest experience. (PDF) [file pone.0214155.s013.pdf]

Table S8: Difference in Outcomes for Induced and Self-Selected Innovators by GPA Controls

	(1) Submission	(2) Average Ranking	(3) Average Ranking Conditional on Submitting
Induced	-0.064	-0.487	-1.978
	(0.069)	(0.277)	(1.102)
Above Median CGPA	0.081	0.088	-3.014
	(0.094)	(0.378)	(1.581)
Above Median CGPA*	0.074	0.551	2.314
Induced	(0.093)	(0.373)	(1.470)
Observations	172	172	17
R-squared	0.040	0.065	0.627
Mean dep var	0.09	0.332	3.715

Notes: Standard errors are in parentheses. All columns include controls for participant gender, cgpa, year of study, whether or not they major in computer science or electrical engineering, and whether or not they have prior innovation contest experience.
