# Supplementary material for: Increasing STEM undergraduate participation in innovative activities: Field experimental evidence
Source: PLoS One. 2019 Apr 5;14(4):e0214155. doi: 10.1371/journal.pone.0214155 (PMC6450611; doi:10.1371/journal.pone.0214155)
Supplement: S9 Table — Standard errors are in parentheses. * significant at 10%; ** significant at 5%; *** significant at 1%. (PDF) [file pone.0214155.s014.pdf]

Table S9: Effect of Encouragement Treatment by GPA

	(1) Submission	(2) Average Ranking	(3) Average Ranking Conditional on Submitting
Encouragement	0.052	0.397	1.890*
	(0.064)	(0.256)	(0.905)
Above Median CGPA	0.052	0.246	0.675
	(0.059)	(0.237)	(0.855)
Above Median CGPA*	-0.139*	-0.827**	-4.510***
Encouragement	(0.084)	(0.338)	(1.344)
Constant	0.073	0.228	3.110***
	(0.045)	(0.180)	(0.715)
Observations	190	190	17
R-squared	0.018	0.037	0.529
Mean dep var	0.09	0.332	3.715

Notes: Standard errors are in parentheses. * significant at 10%; ** significant at 5%; *** significant at 1%
