# Supplementary material for: Increasing STEM undergraduate participation in innovative activities: Field experimental evidence
Source: PLoS One. 2019 Apr 5;14(4):e0214155. doi: 10.1371/journal.pone.0214155 (PMC6450611; doi:10.1371/journal.pone.0214155)
Supplement: S10 Table — Tandard errors are in parentheses. All columns include controls for participant gender, year of study, whether or not they major in computer science or electrical engineering, and whether or not they have prior innovation contest experience. (PDF) [file pone.0214155.s015.pdf]

Table S10: Effect of Encouragement Treatment by GPA, Controls

	(1) Submission	(2) Average Ranking	(3) Average Ranking Conditional on Submitting
Encouragement	0.067	0.477	2.490
	(0.068)	(0.273)	(1.414)
Above Median CGPA	0.202	0.831	1.619
	(0.096)	(0.387)	(2.239)
Above Median CGPA*	-0.163	-0.920	-4.488
Encouragement	(0.092)	(0.371)	(1.667)
Observations	172	172	17
R-squared	0.054	0.081	0.726
Mean dep var	0.09	0.332	3.715

Notes: Standard errors are in parentheses. All columns include controls for participant gender, cgpa, year of study, whether or not they major in computer science or electrical engineering, and whether or not they have prior innovation contest experience.
