# Supplementary material for: Increasing STEM undergraduate participation in innovative activities: Field experimental evidence
Source: PLoS One. 2019 Apr 5;14(4):e0214155. doi: 10.1371/journal.pone.0214155 (PMC6450611; doi:10.1371/journal.pone.0214155)
Supplement: S11 Table — Standard errors are in parentheses. * significant at 10%; ** significant at 5%; *** significant at 1%. (PDF) [file pone.0214155.s016.pdf]

Table S11: Difference in Outcomes for Induced and Self-Selected Participants by Encouragement and GPA

	(1) Submission	(2) Average Ranking	(3) Average Ranking Conditional on Submitting
Induced	0.028	-0.106	-2.835*
	(0.092)	(0.366)	(1.494)
Encouragement	0.152	0.776**	0.083
	(0.096)	(0.382)	(1.364)
Above Median CGPA	0.055	0.101	-1.543
	(0.085)	(0.339)	(1.364)
Encouragement *	-0.191	-0.742	2.418
Induced	(0.129)	(0.515)	(2.023)
Above Median CGPA*	0.000	0.314	3.600*
Induced	(0.121)	(0.484)	(1.761)
Above Median CGPA*	-0.235**	-1.140**	-2.541
Encouragement	(0.118)	(0.474)	(1.929)
Above Median CGPA*	0.177	0.563	-2.853
*Induced	(0.171)	(0.683)	(2.818)
Constant	0.059	0.294	5.000***
	(0.069)	(0.278)	(1.220)
Observations	190	190	17
R-squared	0.036	0.068	0.684
Mean dep var	0.0895	0.332	3.715

Notes: Standard errors are in parentheses. * significant at 10%; ** significant at 5%; *** significant at 1%
