# Supplementary material for: Increasing STEM undergraduate participation in innovative activities: Field experimental evidence
Source: PLoS One. 2019 Apr 5;14(4):e0214155. doi: 10.1371/journal.pone.0214155 (PMC6450611; doi:10.1371/journal.pone.0214155)
Supplement: S12 Table — Standard errors are in parentheses. All columns include controls for participant gender, cgpa, year of study, whether or not they major in computer science or electrical engineering, and whether or not they have prior innovation contest experience. (PDF) [file pone.0214155.s017.pdf]

Table S12: Survey Outcomes by Induced Participants and Encouragement Treatment

	(1)<br>Any Effort on Contest	(2)<br>No Submission due to Time	(3)<br>No Submission due to Challenge
Induced	-0.040	-0.058	0.023
	(0.122)	(0.152)	(0.154)
Encouragement	-0.081	0.304**	0.004
	(0.119)	(0.143)	(0.145)
Observations	72	59	59
R-squared	0.063	0.124	0.066
Mean dep var	0.684	0.470	0.409

Notes: Standard errors are in parentheses. All columns include controls for participant gender, cgpa, year of study, whether or not they major in computer science or electrical engineering, and whether or not they have prior innovation contest experience.
